# Comparison of Coronary Artery Bypass Grafting Combined With Mitral Valve Repair Versus Coronary Artery Bypass Grafting Alone in Patients With Moderate Ischemic Mitral Regurgitation: A Meta-Analysis

**DOI:** 10.7759/cureus.37238

**Published:** 2023-04-07

**Authors:** Muhammad Ali Sameer, Bilal Aziz Malik, Muhammad Obaid Ullah Choudry, Muhammad Shoaib Anwar, Muhammad A Nadeem, Fizza Mahmood, Muhammad Zohaib Anwar, Sujith K Palleti

**Affiliations:** 1 Internal Medicine, CMH Lahore Medical College and Institute of Dentistry, Lahore, PAK; 2 Pharmacology and Therapeutics, CMH Lahore Medical College and Institute of Dentistry, Lahore, PAK; 3 Medicine and Surgery, Shifa International Hospital Islamabad, Islamabad, PAK; 4 Cardiology/Cardiac Surgery, Shifa College of Medicine, Islamabad, PAK; 5 Internal Medicine, Aga Khan University, Karachi, PAK; 6 Nephrology, Edward Hines Jr. Veterans Administration Hospital, Hines, USA; 7 Nephrology, Loyola University Medical Center, Maywood, USA

**Keywords:** meta-analysis, mortality, mitral valve repair, coronary artery bypass, ischemic mitral regurgitation

## Abstract

The aim of this meta-analysis was to compare clinical outcomes between those who underwent coronary artery bypass grafting (CABG) alone and CABG with mitral valve repair (MVR) in patients with moderate ischemic mitral regurgitation. The present study was conducted following the Preferred Reporting Items for Systematic Reviews and Meta-Analyses (PRISMA) guidelines. Two authors performed a comprehensive search of international databases, including PubMed, EMBASE, and the Cochrane Library, for relevant studies published from inception to March 1, 2023. The search was performed again before the submission of the manuscript on March 20, 2023. Primary outcomes assessed in the present meta-analysis included early mortality and long-term mortality. Secondary outcomes assessed in the present meta-analysis included change in New York Heart Association (NYHA) score from baseline, change in ejection fraction (EF) from baseline (%), and major cardiovascular events (MACE). A total of 13 studies were included in the present meta-analysis. Out of 13 included studies, four were randomized control trials (RCTs) and nine were retrospective cohort studies.

The pooled analysis showed that early mortality was significantly lower in patients in the CABG group compared to the CABG+MVR group (risk ratio [RR]: 0.47, 95% confidence interval [CI]: 0.31, 0.70). Long-term mortality was also lower in patients who underwent CABG compared to patients in the CABG+MVR group. However, the difference was statistically insignificant (RR: 0.88, 95% CI: 0.77, 1.02). No significant differences were reported in the EF score between patients who underwent CABG and patients who underwent CABG plus MVR (mean difference [MD]: 0.40, 95% CI: -1.90, 2.69). NYHA score was significantly lower in patients in the CABG+repair group compared to the CABG alone group (MD: 0.39, 95% CI: 0.06, 0.72).

In conclusion, our meta-analysis suggests that concomitant MVR during CABG may not improve clinical outcomes in patients with moderate ischemic mitral regurgitation. Further clinical trials are needed to investigate this intervention in more detail.

## Introduction and background

Ischemic mitral regurgitation of moderate severity occurs in nearly 10% of patients after myocardial infarction [1]. It is caused by the shifting of papillary muscles, decreased closing forces, leaflet tethering, and annular dilatation. Over time, the illness can lead to adverse effects on cardiovascular outcomes [2]. Ischemic mitral regurgitation is frequently observed in patients with multivessel coronary artery disease that requires surgical intervention, raising questions about the necessity of mitral valve intervention during coronary artery bypass grafting (CABG), and the optimal treatment approach has not been well-defined in the medical literature [3]. Although ischemic mitral regurgitation is linked to unfavorable outcomes following CABG, the effect of mitral valve intervention during CABG is a matter of dispute among cardiologists and cardiovascular surgeons [4].

Currently, there is general agreement regarding the treatment of severe ischemic mitral regurgitation. However, the most efficient approach for the treatment of moderate ischemic mitral regurgitation remains controversial [4]. Some experts believe that revascularization alone for moderate ischemic mitral regurgitation, due to improvements in global and regional left ventricular function and geometry after CABG, can decrease rates of mitral regurgitation [5,6]. On the other hand, some experts support restrictive mitral annuloplasty repair at the time of CABG to decrease the degree of mitral regurgitation, preventing further adverse remodeling and reducing the risk of heart failure [7,8]. Nonetheless, incorporating mitral valve repair (MVR) into CABG requires exposing the heart through open-heart surgery, resulting in a longer period of aortic cross-clamping and cardiopulmonary bypass, both of which can raise the risk of complications during the perioperative period [9].

It remains uncertain whether the reduced occurrence of mitral regurgitation after the combined procedure confers any clinical advantages. Several studies have indicated that simultaneous mitral valve surgery results in functional improvements [8, 10], while others have found no benefits in terms of symptoms or survival associated with the incorporation of mitral valve surgery into CABG [11,12]. Several new studies have been conducted since the last meta-analysis that compared the survival and cardiologic outcomes of patients who underwent CABG alone versus CABG with MVR for those with moderate ischemic mitral regurgitation [13]. Therefore, this updated meta-analysis has been conducted to compare clinical outcomes between those who underwent CABG alone and CABG with MVR in patients with ischemic mitral regurgitation.

## Review

Methodology

The present study was conducted following the Preferred Reporting Items for Systematic Reviews and Meta-Analyses (PRISMA) guidelines.

Search Strategy and Study Selection

Two authors performed a comprehensive search of international databases, including PubMed, EMBASE, and the Cochrane Library, for relevant studies published from inception to March 1, 2023. The search was performed again before the submission of the manuscript on March 20, 2023. The search strategy used Medical Subject Headings (MeSH) terms and Boolean algebra operators to achieve maximum sensitivity. The keywords used for the search were “ischemic mitral regurgitation,” “coronary artery bypass,” and “mitral valve surgery.” The reference lists of all included articles were also reviewed to prevent any article from being missed in the meta-analysis.

The study selection process was conducted independently by two authors. In the first level, titles and abstracts were reviewed, and the full text of eligible studies was obtained for a detailed assessment of inclusion and exclusion criteria. Any disagreement between the two authors in the process of study selection was resolved through discussion.

Eligibility Criteria

The present meta-analysis included all studies that involved adult patients with clinically significant coronary artery disease and associated ischemic mitral regurgitation. Only those studies that directly compared outcomes between patients who underwent CABG only and those who underwent CABG with MVR were included in the analysis. Case reports, case series, and review articles were excluded from the study. Additionally, studies that did not report the outcomes assessed in the present meta-analysis were also excluded. No limitations were imposed on the sample sizes and patient characteristics.

Data Extraction, Outcomes Measures and Quality Assessment

The data extraction of each included study was carried out using an Excel-based sheet. Data extraction was performed by two authors independently. Any disagreement between two authors was resolved by consensus or discussion with the third author. The data extracted from individual studies included author name, year of publication, study groups, sample size, follow-up duration and patients’ characteristics.

Primary outcomes assessed in the present meta-analysis included early mortality (including in-hospital and mortality within 30 days of the intervention) and long-term mortality. Secondary outcomes assessed in the present meta-analysis included change in NYHA (New York Heart Association) score from baseline, change in ejection fraction (EF) from baseline (%) and major cardiovascular events (MACE). The quality of the studies was independently assessed by two authors independently using the Cochrane quality assessment tool for randomized controlled trials (RCTs) and the New Castle-Ottawa Scale (NCOS) for observational studies.

Statistical Analysis

Analysis was performed using Review Manager (RevMan) Version 5.4.1. (The Nordic Cochrane Centre, the Cochrane Collaboration, Denmark). Outcomes were assessed using standard techniques of meta-analysis. The mean difference (MD) was calculated for continuous outcomes with 95% confidence interval (CI) and risk ratio (RR) was reported for categorical outcomes with their 95% CI. Summary statistics were calculated using fixed-effect or random-effect models based on I-square statisics calculation. Pooled RR and mean differences were calculated using fixed effect model if I-square statistics were <50%. Otherwise random-effect model was used to calculate pooled estimates. A p-value less than 0.05 were considered significant. I-square value of less than 25% represented mild heterogeneity, 25%-50% represented moderate heterogeneity and higher than 50% represented high heterogeneity. Meta-analysis results are displayed in forest plots. Subgroup analysis was performed based on study design including observational studies and RCTs.

Results

**Figure 1:**
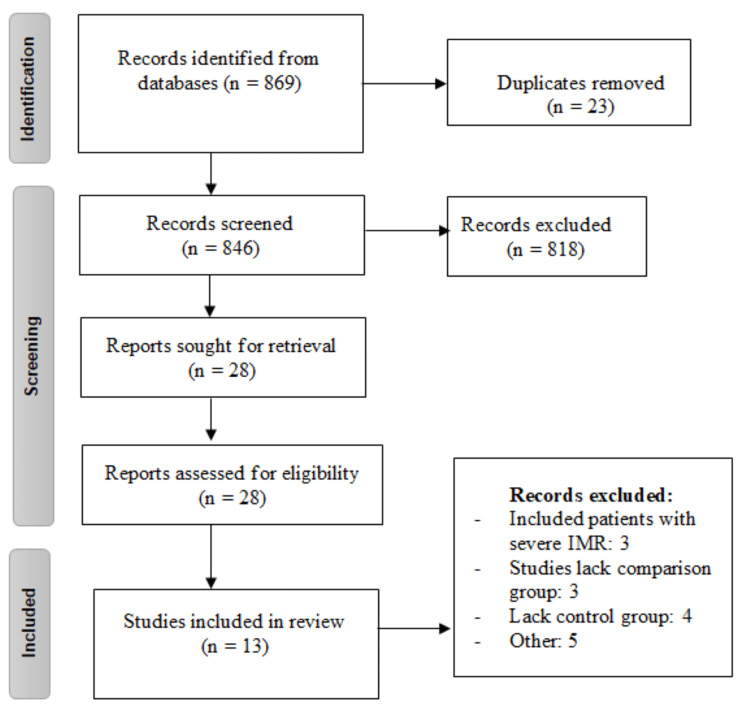
PRISMA flowchart of selection of studies

**Figure 2:**
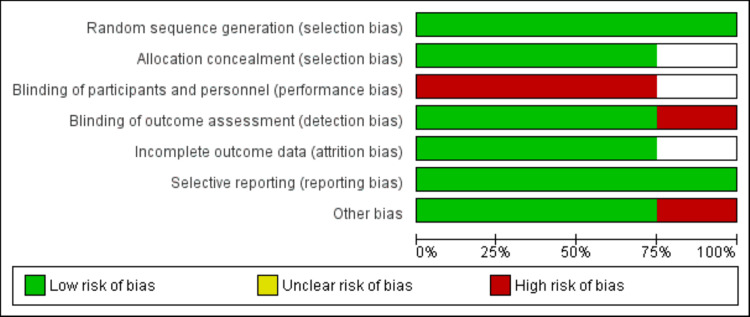
Quality assessment of RCTs

Our systematic search yields 869 articles. After removing duplicates, 846 studies were gone through title and abstract screening. After the screening process, 818 studies were excluded and full text of remaining 28 studies was retrieved for detailed assessment of inclusion and exclusion criteria. Finally, 13 studies were included in the present meta-analysis. Figure 1 shows the process of selection of studies. Table 1 shows characteristics of included studies. Out of 13 included studies, four were RCTs and nine were retrospective cohort. In all included studies, majority of the participants were males. Figure 2 shows quality assessment of all included RCTs, while observational studies quality assessment is shown in Table 2.

**Table 1:** Characteristics of included studies RCT: Randomized controlled trial; CABG: Coronary artery bypass graft; NR: Not reported

Author Name	Year	Design	Setting	Groups	Sample Size	Follow-up	Age (Years)	Male (%)
Bouchard et al [14]	2014	RCT	Single-center	CABG	16	1 Year	65 vs 69	88 vs 75
				CABG+Repair	15			
Chan et al [8]	2012	RCT	Multicenter	CABG	38	1 Year	70.4 vs 70.9	74 vs 74
				CABG+Repair	38			
Fattouch et al [7]	2009	RCT	Single-center	CABG	54	5 Years	66 vs 64	64.8 vs 62.5
				CABG+Repair	48			
Goland et al [15]	2009	Retrospective	Single-center	CABG	55	5 Years	69 vs 68	64 vs 80
				CABG+Repair	28			
Hamouda et al [16]	2017	Retrospective	Single-center	CABG	69	4 Years	67 vs 63	86.9 vs 85.7
				CABG+Repair	77			
Harris et al [17]	2002	Retrospective	Single-center	CABG	142	5 Years	68.8 vs 65.6	54 vs 44
				CABG+Repair	34			
Kim et al [18]	2018	Retrospective	Single-center	CABG	594	7 Years	64.9 vs 64.7	70.8 vs 69.1
				CABG+Repair	116			
Liu et al [19]	2023	Retrospective	Single-center	CABG	62	1 Year	62.8 vs 57.9	67.7 vs 71.4
				CABG+Repair	21			
Michler et al [9]	2016	RCT	Multicenter	CABG	151	2 Years	65.2 vs 64.3	65.6 vs 70.7
				CABG+Repair	150			
Rilinger et al [20]	2018	Retrospective	Single-center	CABG	21	5 Years	71 vs 69	67 vs 81
				CABG+Repair	21			
Totkas et al [21]	2016	Retrospective	Single-center	CABG	46	1 Year	63 vs 61	52.8 vs 58.7
				CABG+Repair	44			
Trichon et al [22]	2003	Retrospective	Single-center	CABG	687	1 Year	68 vs 68	53 vs 52.2
				CABG+Repair	228			
Wong et al [12]	2005	Retrospective	Single-center	CABG	220	5 Years	NR	NR
				CABG+Repair	31			

**Table 2:** Quality assessment of observational studies

Study ID	Selection	Comparibility	Outcome	Overall
Goland et al [15]	4	1	2	Good
Hamouda et al [16]	3	2	2	Good
Harris et al [17]	3	1	2	Fair
Kim et al [18]	4	2	3	Good
Liu et al [19]	3	1	2	Good
Rilinger et al [20]	4	2	3	Good
Totkas et al [21]	3	1	2	Fair
Trichon et al [22]	2	2	2	Fair
Wong et al [12]	4	1	2	Good

Meta-analysis of outcomes

Early Mortality and Long-Term Mortality

**Figure 3:**
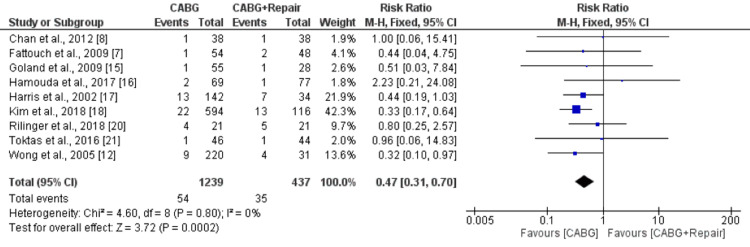
Forest plot comparing risk of mortality between two groups CABG: Coronary artery bypass surgery Sources: References [7,8,12,15-18,20,21]

**Figure 4:**
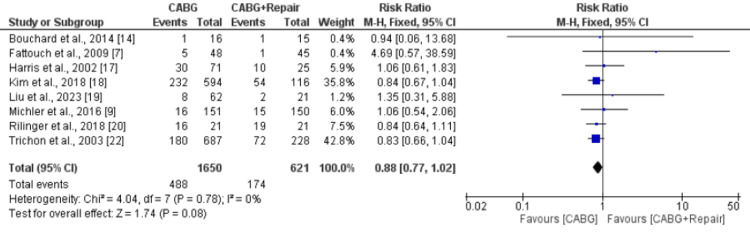
Forest plot comparing risk of long-term mortality between two groups CABG: Coronary artery bypass surgery Sources: References [7,9,14,17-20,22]

A pooled analysis of nine studies, including 1,676 patients, showed that early mortality was significantly lower in patients in the CABG group compared to the CABG+MVR group (RR: 0.47, 95% CI: 0.31, 0.70), as shown in Figure 3. No significant heterogeneity was reported among the study results (I-squared: 0%, p-value: 0.80). Long-term mortality was also lower in patients who underwent CABG compared to patients in the CABG+MVR group. However, the difference was statistically insignificant (RR: 0.88, 95% CI: 0.77, 1.02), as shown in Figure 4. No significant heterogeneity was reported among the study results (I-squared: 0%, p-value: 0.78).

**Figure 5:**
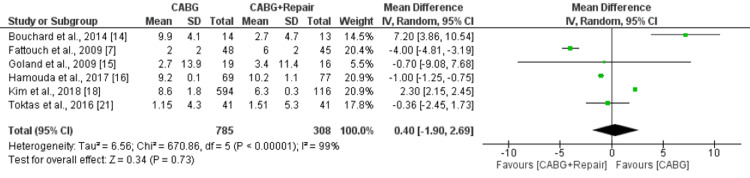
Forest plot comparing EF between two groups CABG: Coronary artery bypass surgery Sources: References [7,14-16,18,21]

Six studies comprising 1093 patients were included in our analysis. No significant differences were reported in the EF score between patients underwent CABG and patients underwent CABG plus MVR (MD: 0.40, 95% CI: -1.90, 2.69) as shown in Figure 5. High heterogeneity was reported among the study results (I-square: 99%, p-value<0.0001).

**Figure 6:**
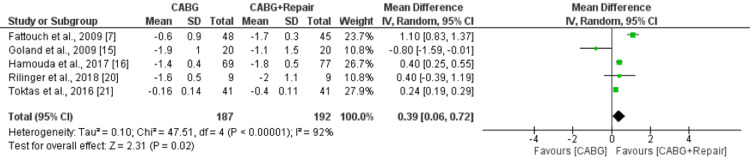
Forest plot comparing NYHA score between two groups CABG: Coronary artery bypass surgery Sources: References [7,15,16,20,21]

Six studies comprising 379 patients were included in the pooled analysis. Overall, NYHA score was significantly lower in patients in CABG+repair group compared to the CABG along group (MD: 0.39, 95% CI: 0.06, 0.72) as shown in Figure 6. High heterogeneity was reported among the study results (I-square: 92%, p-value<0.0001).

Three studies involving 1094 patients were included in our pooled analysis. No significant difference was found between two groups in the risk of MACE events (RR: 0.94, 95% CI: 0.66, 1.33). No significant heterogeneity was reported among the study results (I-square: 0%, p-value: 0.63).

Subgroup Analysis

Table 3 shows the results of the subgroup analysis. Regarding early mortality and long-term mortality, no significant differences were found between the two groups in the pooled analysis of RCTs. However, analysis of observational studies revealed higher early and long-term mortality in patients in the CABG plus repair group. Results of NYHA were consistent across subgroups (observational and RCTs).

**Table 3:** Results of subgroup analysis NYHA: New York Heart Association Functional Classification; EF: Ejection fraction; RR: Risk ratio; CI: Confidence interval; RCT: Randomized controlled trial ^ Presented as mean difference (95% CI) * Significant at p-value<0.05

Outcomes	Subgroups	Number of studies	RR (95% CI)	I-square
Early Mortality	RCT	2	0.62 (0.11, 3.61)	0%
	Observational	7	0.46 (0.30, 0.69)*	0%
Long term Mortality	RCT	3	1.23 (0.62, 2.44)	0%
	Observational	5	0.86 (0.74-0.99)*	0%
NYHA^	RCT	1	1.10 (0.83-1.37)*	NA
	Observational	4	0.25 (0.04, 0.46)*	72%
EF (%)^	RCT	2	1.48 (-9.49, 12.45)	54%
	Observational	4	0.28 (-2.32, 2.88)	69%

Discussion

In this meta-analysis, we evaluated the effectiveness of concomitant mitral valve (MV) repair during CABG in improving clinical outcomes in patients with moderate ischemic mitral regurgitation. Our findings showed that while there were no significant differences in long-term mortality between the two groups, short-term mortality was significantly higher in patients who received concomitant MV repair during CABG surgery. We conducted subgroup analyses based on study design, and the pooled analysis of RCTs did not report any significant differences between the two study groups in terms of early or long-term mortality. However, the pooled analysis of observational studies showed higher early mortality and long-term mortality in patients who underwent CABG with MVR, primarily due to the high weightage of three studies [12,17,18]. Conversely, all other studies did not report any significant differences in early mortality between the two groups. Although long-term mortality was higher in patients who underwent CABG with MV repair, the difference was insignificant, with only two out of eight studies reporting a significantly higher risk in these patients compared to their counterparts [18,22]. Finally, the pooled analysis of observational studies reported a significantly higher risk in patients who underwent CABG with MV repair, primarily due to the high weightage of two studies [18,22].

Compared with CABG alone, CABG plus MVR improved heart failure symptoms, including changes in the NYHA class baseline, but no significant differences were reported between the two groups in terms of change in EF. The meta-analysis conducted by Kopjar et al. did not report any significant differences between the two groups in NYHA class [14]. However, our study assessed the change in NYHA score from baseline instead of comparing the number of patients with NYHA functional class ≥2 at follow-up.

The RCT conducted by Fattouch et al. [7], which compared isolated CABG with CABG plus MVR in patients with moderate ischemic mitral regurgitation, found a greater reduction in NYHA score in CABG plus MVR patients compared to patients who underwent CABG alone. Similarly, in the RIME study conducted by Chan et al. [10], patients who underwent CABG plus MV repair showed a greater improvement in their peak oxygen consumption after one year compared to those who only underwent CABG (22% vs. 5%). This improvement was also reflected in the lower NYHA functional class among patients who received both CABG and MV repair simultaneously.

The decision to operate on the mitral valve for IMR depends on the severity of the regurgitation and whether or not CABG is necessary. For patients with moderate IMR who require CABG, some surgeons recommend MV surgery to improve symptom relief. However, there is limited evidence to support this approach. The rationale is that CABG alone may not effectively reduce regurgitation, and persistent or worsening MR may lead to poor outcomes. On the other hand, proponents of isolated CABG argue that treating the underlying cause can result in reverse remodeling of the left ventricle, which can reduce MR. The effectiveness of this method largely depends on whether viable myocardium is present. Penicka and colleagues showed that patients with moderate IMR who underwent isolated CABG and had viable myocardium and no papillary muscle dyssynchrony had limited improvement in regurgitation [23].

Castleberry et al. conducted the most extensive research to date on the management of ischemic mitral regurgitation [24]. Their study was a retrospective analysis conducted in a single center. The research team reviewed medical records of 4,989 patients with moderate to severe ischemic mitral regurgitation over a decade. The study participants were treated with medication, percutaneous coronary intervention, CABG, or both CABG and mitral valve surgery. The results showed that isolated CABG had the highest adjusted survival rate after ten years [24].

Study limitations

In our meta-analysis, some reports utilized outdated surgical techniques, and there have been significant improvements in the techniques for MVR and types of annuloplasty prostheses over time. Although some observational studies included in the analysis reported outcomes of patients who underwent surgery over 20 years ago, a separate analysis of only RCT data strengthened our results. These trials included patients from a recent period who followed current principles of MV repair. Furthermore, the present meta-analysis revealed higher rates of early and long-term mortality among patients receiving CABG alone, largely due to the significant weighting of particular studies. As these studies were of an observational nature, caution should be exercised in interpreting the results. Moreover, definitions of some clinical endpoints differed slightly across studies, which may have reduced the accuracy of our findings. Moreover, significant heterogeneity was reported for EF and NYHA outcomes. This heterogeneity might be attributed to significant variation in the follow-up duration and differences in the evaluation of these scores. To account for heterogeneity among studies, we used a random-effect model. However, since only four RCTs were conducted on this topic, more multicenter RCTs need to be conducted with a large sample size in the future to obtain more accurate findings that can help healthcare professionals develop recommendations regarding when and how to carry out MVR while performing CABG.

## Conclusions

In conclusion, our meta-analysis suggests that concomitant MVR during CABG may not improve clinical outcomes in patients with moderate ischemic mitral regurgitation. Our findings demonstrate that early mortality was significantly lower in patients who underwent CABG alone compared to those who received CABG with MVR. Although long-term mortality was also lower in the CABG alone group, the difference was statistically insignificant. The analysis of ejection fraction showed no significant difference between the two groups. However, the analysis of the New York Heart Association Functional Classification showed that patients in the CABG plus repair group had a significantly lower score than those in the CABG alone group. The subgroup analysis based on study design showed that the pooled analysis of randomized controlled trials did not report any significant differences between the two groups in terms of early or long-term mortality, while the pooled analysis of observational studies showed higher early and long-term mortality in patients who underwent CABG with MVR. Overall, the results suggest that careful consideration should be taken when deciding whether to perform concomitant MVR during CABG surgery. Further studies are needed to investigate this issue in more detail.
